# Clinical efficacy of Danshen preparation in the treatment of vascular cognitive impairment: A systematic review and meta-analysis

**DOI:** 10.3389/fnagi.2022.1090665

**Published:** 2023-01-20

**Authors:** Yunze Li, Yangjing Yao, Xinran Cao, Nan Yi, Anqi Chen, Jianjun Li, Minghua Wu

**Affiliations:** ^1^Jiangsu Provincial Hospital of Chinese Medicine, Affiliated Hospital of Nanjing University of Chinese Medicine, Nanjing, China; ^2^Nanjing University of Chinese Medicine, Nanjing, China; ^3^Lianyungang Hospital of Traditional Chinese Medicine, Lianyungang Affiliated Hospital of Nanjing University of Chinese Medicine, Lianyungang, China

**Keywords:** Danshen preparation, vascular cognitive performance, clinical studies, systematic review, meta-analysis

## Abstract

**Ethnopharmacological relevance:**

Danshen preparations are widely used in the treatment of ischaemic cerebrovascular disease. However, the clinical efficacy of such preparations remains unclear. Consequently, Danshen preparations are used to a lesser extent in vascular cognitive impairment (VCI).

**Aim of the study:**

In this study, we aimed to systematically assess the clinical efficacy and safety of Danshen preparations in VCI. To this end, we examined and performed a meta-analysis (MA) of the evidence available from randomised controlled trials (RCTs) of Danshen preparations conducted in patients with VCI.

**Methods:**

We queried the following sources and collected all articles reporting on RCTs of Danshen preparations published prior to December 2021: PubMed, China National Knowledge Infrastructure (CNKI), Wanfang Data, Chongqing VIP Database (CQVIP), and China Biology Medicine (CBM) disc databases. The assessment of treatments that were included in references were performed by RevMan 5.2 software based on guidelines from Cochrane Handbook for Systematic Reviews of Interventions.

**Results:**

We included a total of 12 RCTs that included data on clinical therapeutic effects. The evaluation criteria included the following: National Institutes of Health Stroke Scale (NIHSS), modified Rankin Scale (mRS), Barthel Index (BI), Mini-Mental State Assessment (MMSE), Montreal Cognitive Assessment (MOCA), Activities of Daily Living Scale (ADL), treatment effect index, and incidence of adverse reaction index. In the included studies, the observation groups included 656 cases and the control groups included 660 cases. The results of the MA were as follows: the mean difference (MD) value after combining the effect size for NIHSS was −2.91, with a 95% confidence interval (CI) of −4.22 to −1.59; the combined effect quantity hypothesis test revealed that *Z* = 4.33 (*p* < 0.00001), indicating that the score pertaining to the degree of neurological deficit (NIHSS) in the observation group after treatment was significantly lower than that in the control group. This result reveals that treatment with a Danshen preparation can reduce neurological deficit in VCI patients. The MD value after combining the effect size for mRS was −0.73, with a 95% CI of −0.85 to −0.61; the result of the combined effect quantity hypothesis test revealed that *Z* = 12.29 (*p* < 0.00001). These results indicate that the degree of disability was significantly reduced after treatment in the observation group. The MD value after combining the effect size for MMSE was 2.09, with a 95% CI of 0.33–3.84; the result of the combined effect quantity hypothesis test showed that *Z* = 2.33 (*p* = 0.02). These results indicate that the cognitive function score (MMSE) of the observation group after treatment was significantly higher than that of the control group and suggests that the cognitive function of VCI patients was improved after treatment with Danshen preparations. The MD value after combining the effect size for ADL was 8.79, with a 95% CI of 3.52 to 14.06; the result of the combined effect quantity hypothesis test indicated that *Z* = 3.27 (*p* = 0.001). These results showed that the life ability (ADL scale) scores of patients in the observation group after treatment were significantly higher than those in the control group, and reveals that after treatment with Danshen preparations, patients exhibited significant improvement in life ability. The MD value after combining the effect size for high-sensitivity C-reactive protein (hs-CRP) was −3.21, with a 95% CI of −4.21 to −2.22; the result of the combined effect quantity hypothesis test showed that *Z* = 6.31 (*p* < 0.00001), indicating that the hs-CRP level in the observation group was significantly decreased after treatment. The MD value after combining the effect size for interleukin (IL)-6 was −2.95, with a 95% CI of −3.86 to −2.04; the result of the combined effect quantity hypothesis test showed that *Z* = 6.36 (*p* < 0.00001). These results showed that the IL-6 level in the observation group was significantly decreased after treatment.

**Conclusion:**

The existing clinical evidence shows that Danshen preparations exert significant therapeutic effects on VCI patients and can ameliorate inflammatory conditions in these patients. In addition, Danshen preparations are relatively safe.

## 1. Introduction

Cerebrovascular disease is one of the major diseases that leads to human fatalities. Haemorrhagic brain damage and ischaemic brain damage lead to different degrees of advanced neurocognitive dysfunction (Zhang, 2012). With the ageing of the population in China, the incidence of vascular cognitive impairment (VCI) is increasing annually, and exerts a significant burden on the society and family. Ischaemic cerebrovascular disease is the most common amongst the VCI diseases, and ameliorating cognitive dysfunction caused by this disease is one of the main challenges faced by neurologists. Continuous apoptosis of brain cells, abnormal cerebral vascular microcirculation, and a lack of proper blood circulation have been implicated in the pathophysiology of VCI (Han et al., 2014). Anticholinergic drugs are clinically applied to treat VCI, though this practice has not gained universal acceptance. The key mode of treatment in VCI aims to promote cerebral vasodilation, increase blood oxygen content, and promote neuronal viability (Guo et al., 2013; Wang H. R. et al., 2014).

Traditional Chinese medicine has achieved remarkable curative effects in VCI patients as evidenced by multi-level and multi-dimensional studies (Hou et al., 2009). Danshen preparations have been frequently used in this regard. In recent years, a number of studies have shown that Danshen preparations have multiple biological activities including natural antioxidant properties, scavenging of a variety of oxygen free radicals, and activation of antagonists of platelet-activating factor. Danshen preparations can also significantly dilate cerebral vessels and improve the symptoms of cerebral ischaemia and hypoxia. The active ingredients in Danshen preparations have been shown to have a variety of neuroprotective and restorative effects which help maintain the blood–brain barrier, reduce cerebral oedema, improve energy metabolism, exert antioxidant, anti-inflammatory, and anti-apoptotic effects, promote angiogenesis, and exert therapeutic effects in VCI. Danshen preparations have been applied in various forms including Danshen injections, Danshen tablets, Sodium Tanshinone IIA Sulfonate, Danshen dripping pill, Danshen granules, and Danshen capsules. Tanshinone can promote the proliferation and differentiation of neural stem cells cultured *in vitro* and can also promote the proliferation of endogenous neural stem cells and improve neurological function after ischaemia (Chen et al., 2011).

Danshen preparations are widely used in traditional Chinese medicine and are economical and efficient blood stasis-removing agents (Wu et al., 2015). These preparations have been in use since many years, and their application has shown an increasing trend annually; reports of adverse events have also gradually increased. The most common adverse reactions were allergic reactions and abnormal bleeding. Tanshinone and acidic crystals are amongst the active ingredients in Danshen preparations. These components may function as haptens and cause allergic reactions after binding to plasma proteins in the body. The tannins contained in Danshen preparations cannot be effectively removed by the current methods of preparation. The tannins form macromolecular complexes after binding to the amino groups in plasma proteins, causing allergic reactions. Danshen preparations contain a number of insoluble particles that may occlude local blood vessels, which frequently leads to phlebitis and thromboembolic events (Gong, 2021).

In this study, we retrieved all publications (published prior to December 2021) reporting on RCTs of Danshen preparations used in the treatment of VCI; we aimed to evaluate the clinical efficacy and safety of Danshen preparations in the treatment of VCI, and provide references for the clinical treatment of VCI.

## 2. Methods

We performed a MA according to the Cochrane Handbook for Systematic Reviews of Interventions (Shuster, 2011); the results are presented as per the Preferred Reporting Items for Systematic Reviews and Meta-Analyses guidelines (Moher et al., 2009). Our MA has been registered on PROSPERO (registration number: CRD42022299993). The research followed guidelines of the Declaration of Helsinki and Tokyo for humans, and was approved by the institutional human experimentation committee or equivalent, and that informed consent was obtained, ethics review approval document no. 2022NL-010-02.

### 2.1. Literature search

The following databases were queried by two researchers (Yunze Li and Yangjing Yao): PubMed, China National Knowledge Infrastructure (CNKI), Wanfang Data, Chongqing VIP Database (CQVIP), and China Biology Medicine (CBM) disc databases. All publications pertaining to RCTs on Danshen preparations published prior to December 2021 were retrieved. The following search terms were used (applying pseudo-logic): [VCI or vascular dementia (VD) or post-stroke dementia] AND (Danshen or tanshinone or salvianolic acid or Danshen preparation) AND (randomised controlled trial).

### 2.2. Inclusion and exclusion criteria

We applied the Participants, Interventions, Comparisons, Outcomes and Study Design (PICOS) approach to establish our inclusion criteria, which were as follows: (1) patients must meet the accepted diagnostic criteria for VCI, regardless of sex, age, body mass index (BMI), or other indicators; (2) Danshen preparations are compared with other interventions; the comparisons may include Danshen preparations plus conventional treatment versus conventional treatment; and (3) studies that report results pertaining to at least one of the following: National Institutes of Health Stroke Scale (NIHSS), clinical treatment effects, Barthel Index (BI), adverse reactions, and inflammatory factors.

The exclusion criteria were as follows: (1) publications such as reviews, experiences, case reports, conference reports, and animal experiments; (2) duplicate publications, duplicate data, and incomplete literature; (3) reports on medical interventions using other traditional Chinese medicine modalities, Chinese proprietary medicines, acupuncture and massage, etc.; (4) publications with <20 cases in the observation group or control group or <40 cases in total.

No language restrictions were imposed.

### 2.3. Research methods

Our reviewers worked in pairs. The reviewers first identified articles that met the inclusion criteria by title and abstract, and then independently verified the full text.

### 2.4. Data collection process

Two researchers (Xinran Cao and Nan Yi) independently extracted the following information using predesigned tables: main authors, year of publication, details of intervention measures, sample size, age of participants, outcomes, and adverse events. In the event of disagreement between the two reviewers in a pair, inclusion or exclusion of the corresponding publication was decided by a third author or by group discussion.

### 2.5. Assessment of risk of bias

The methodological quality of the included studies was assessed by two researchers (Yunze Li and Nan Yi) as per the Cochrane Handbook 5.1.0 (Shuster, 2011). Assessment of the risk of bias was performed according to seven items: random sequence generation, allocation concealment, blinding of investigators and subjects, blinded assessment of the study results, completeness of the outcome data, selective reporting of the study outcomes, and other indicators of bias. The articles were coded as green, yellow, or red with “+,” “−”, and “?” symbols which indicated “low risk of bias,” “high risk of bias”, and “unclear,” respectively. The included articles were evaluated individually. Disagreements were resolved through discussions with a third researcher (Jianjun Li).

### 2.6. Statistical analysis

We used RevMan 5.2 software to process and analyse the extracted data. Binary data were analysed using risk ratios (RR) and continuous variables were analysed using mean differences (MD) and 95% confidence intervals (CI). The *I*^2^ statistic is used to assess heterogeneity amongst the studies. *I*^2^ < 50% and *p* ≥ 0.1 indicated acceptable heterogeneity, and a fixed-effects model was applied for analysis in such cases. *I*^2^ > 50% or *p* ≤ 0.1 indicated significant heterogeneity, and in such cases, a random-effects model was applied to analyse the potential sources of the heterogeneity. Subgroup analyses or sensitivity analyses were performed when necessary. A *p* statistic of <0.05 was considered to indicate a statistically significant result. When more than 10 articles included in the outcome measure, a funnel plot was applied to determine whether publication bias was present.

## 3. Results

### 3.1. Literature retrieval

**Figure 1:**
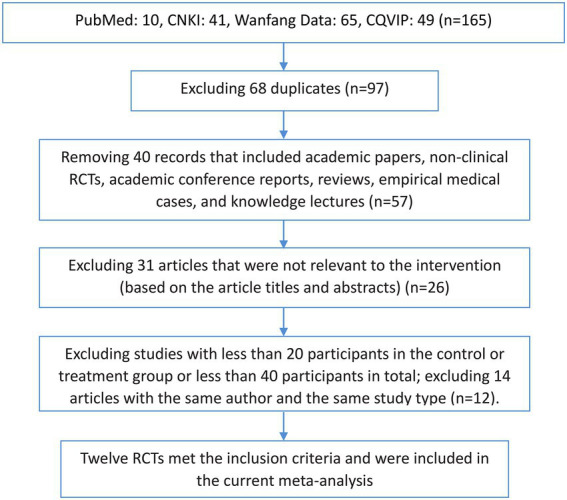
Flow chart of the literature screening process. CNKI, China National Knowledge Infrastructure; CQVIP, Chongqing VIP Database; RCT, randomised controlled trial.

A total of 165 published articles were retrieved, including 41 articles from CNKI, 65 articles from Wanfang Data, 49 articles from CQVIP, and 10 articles from PubMed. The articles were imported into the NoteExpress literature management software, and 68 duplicate references were removed. We also excluded 40 articles which included academic papers, nonclinical RCTs, academic conference reports, reviews, empirical medical cases, and knowledge lectures. In addition, after reading the article titles and abstracts, we excluded 31 articles that were irrelevant to the intervention of interest. After excluding studies with <20 participants in the control or treatment group or <40 participants in total, and after excluding 14 articles with the same author and the same study type, the remaining 12 RCTs were included in our MA. The screening process is shown in Figure 1.

### 3.2. Characteristics of the included studies

A total of 12 articles that met our criteria were included in the study. The studies included 656 cases in the observation groups and 660 cases in the control groups. The evaluation indexes used in the studies were as follows: clinical efficacy, five studies; NIHSS score, six studies; mRS score, two studies; BI score, three studies; ADL score, five articles and six studies [In Li et al. (2014), there are two types of disease, calculated from two studies]; MMSE score, eight articles and nine studies; Montreal Cognitive Assessment (MOCA) score, six articles and seven studies; revised Hasegawa Dementia Scale (HDS-R), two studies; high-sensitivity C-reactive protein (hs-CRP), two studies; interleukin (IL)-6, two studies; tumour necrosis factor (TNF)-α, two studies; and adverse reactions, four studies (as shown in Table 1).

**Table 1:** Details from trials.

First author, year	Reference	Intervention		Sample size		Age		Duration	Outcome
		Treatment	Control	Treatment	Control	Treatment	Control		
Li ZW, 2013	Li et al. (2013)	Tanshinone + routine	Routine	30	30	67.3	67.3	2 weeks	MMSE, FAQ
Li MQ, 2014	Li et al. (2014)	Tanshinone + piracetam + Ginkgo leaf tablets + routine	Vinpocetine + piracetam + Ginkgo leaf tablets + routine	106	110	72.3 ± 4.5	72.6 ± 4.4	4 weeks	MOCA, ADL, NPI, adverse reactions
Wang Y, 2014	Wang Y. et al. (2014)	Salvianolic acid + routine	Routine	43	43	62.5 ± 7.3	62.5 ± 7.3	4 weeks	MMSE, MOCA, HDS-R
Dong XL, 2015	Dong et al. (2015)	Salvianolic acid + routine	Xuesetong + routine	55	55	63 ± 8	63 ± 6	2 weeks	MMSE, NIHSS, ADL, IL-6, TNF-α, CRP
Huang XL, 2015	Huang (2015)	Salvianolic acid + routine	Routine	44	44	56.9 ± 4.3	57.4 ± 4.2	2 weeks	BI, MMSE, MOCA, MRS, NIHSS
Su Y, 2015	Sue et al. (2015)	Tanshinone + piracetam + Ginkgo leaf tablets + routine	Vinpocetine + piracetam + Ginkgo leaf tablets + routine	50	50	57.7 ± 5.6	50.7 ± 9.5	6 weeks	MOCA, ADL, EEG
Liu S, 2016	Liu et al. (2016)	Salvianolic acid + routine	Routine	56	56	62.6 ± 3.6	63.1 ± 3.2	4 weeks	Efficacy, TG, HDL, LDL, Hcy
Shan XY, 2016	Shan (2016)	Salvianolic acid + routine	Routine	74	74	61.38 ± 7.82	60.76 ± 7.13	3 weeks	Efficacy, MMSE, NIHSS, ADL
Wang HH, 2016	Wang et al. (2016)	Danshen tablets + donepezil hydrochloride tablets + routine	Donepezil hydrochloride tablets + routine	64	64	63.6 ± 2.2	63.4 ± 2.1	36 weeks	Efficacy, HDS-R, ADL, adverse reactions
Liu HJ, 2017	Liu et al. (2017)	Salvianolic acid + aspirin + atorvastatin + citicoline + routine	Aspirin + atorvastatin + citicoline + routine	43	43	62.4 ± 3.0	62.3 ± 3.1	2 weeks	Efficacy, MMSE,MOCA,NIHSS
Yan BC, 2017	Yan (2017)	Salvianolic acid + routine	Routine	48	48	55.34 ± 5.67	56.16 ± 6.39	2 weeks	BI, MMSE, MOCA, MRS, NIHSS, adverse reactions
Liu XM, 2020	Liu (2020)	Tanshinone + donepezil hydrochloride tablets + acupuncture	Donepezil hydrochloride tablets + acupuncture	43	43	62.07 ± 10.23	62.19 ± 10.41	8 weeks	Efficacy, MMSE, ADL, NIHSS, BI, IL-6, TNF-α, hs-CRP, adverse reactions

### 3.3. Assessment of risk of bias

**Figure 2:**
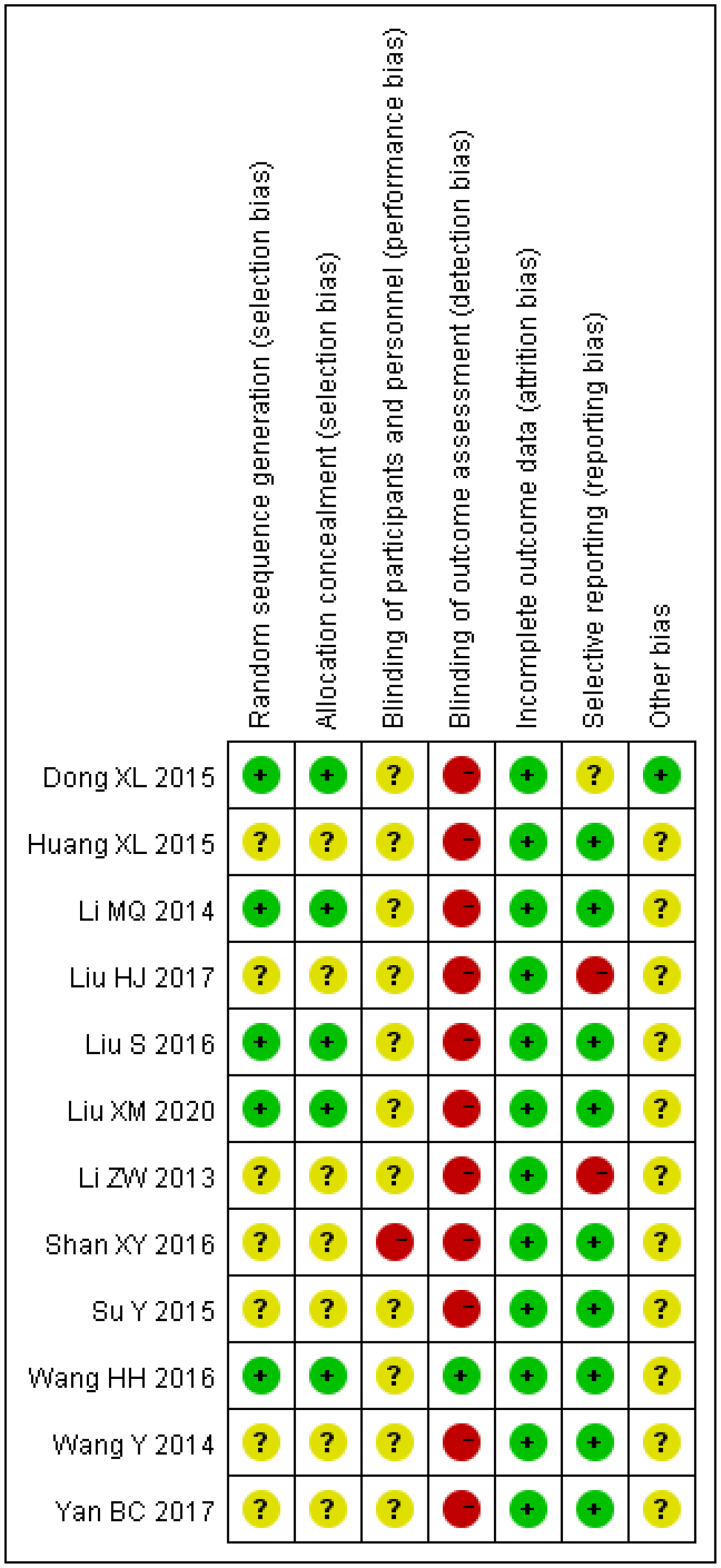
Assessment of risk of bias.

The risk of bias was assessed for all the included studies. All studies had complete data with no missing data, and most studies reported multiple outcome indicators. Therefore, there was less follow-up bias. However, due to the lack of detailed descriptions of randomisation in some studies, it is impossible to determine whether selection or allocation bias was present. In addition, reporting and measurement biases cannot be ignored because most studies did not mention whether the investigators or examiners were blinded (as shown in Figure 2).

### 3.4. Clinical efficacy

Amongst the included studies, a total of five studies assessed clinical efficacy, three studies on salvianolic acid, one study on tanshinone and one study on Danshen tablets, and included 280 patients in the treatment groups and 280 patients in the control groups. The heterogeneity amongst the studies was acceptable (*I*^2^ = 0%, *p* = 0.74), so a fixed-effect model was applied for analysis. After combining the effect size, the results showed that the odds ratio (OR) was 3.22, with a 95% CI of 1.99–5.23; the result of the combined effect quantity hypothesis test indicated a *Z* = 4.75 (*p* < 0.00001). A comparison of the treatment and control groups indicated that the Danshen preparations exerted significant therapeutic effects in the included patient population.


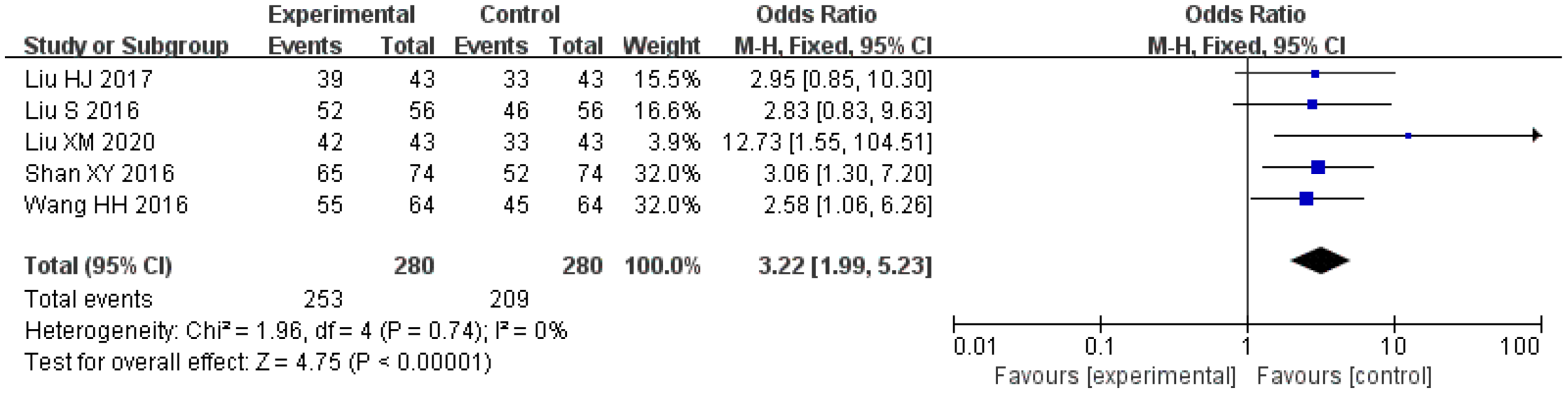


### 3.5. Nervous system scores

#### 3.5.1. NIHSS

Amongst the included studies, the NIHSS was reported in six studies, five studies on salvianolic acid, one study on tanshinone, which included 307 cases in the treatment groups and 307 cases in the control groups. There was significant heterogeneity amongst the studies (*I*^2^ = 96%, *p* < 0.00001). Therefore, a random effects model was applied for analysis. After combining the effect size, the results showed that the MD value was −2.91, with a 95% CI of −4.22 to −1.59; the result of the combined effect quantity hypothesis test was *Z* = 4.33 (*p* < 0.00001). The results indicated that the score pertaining to the degree of neurological deficit (NIHSS) in the observation group after treatment was significantly lower than that in the control group. This demonstrates that treatment with Danshen preparations can reduce neurological deficit in patients with VCI.


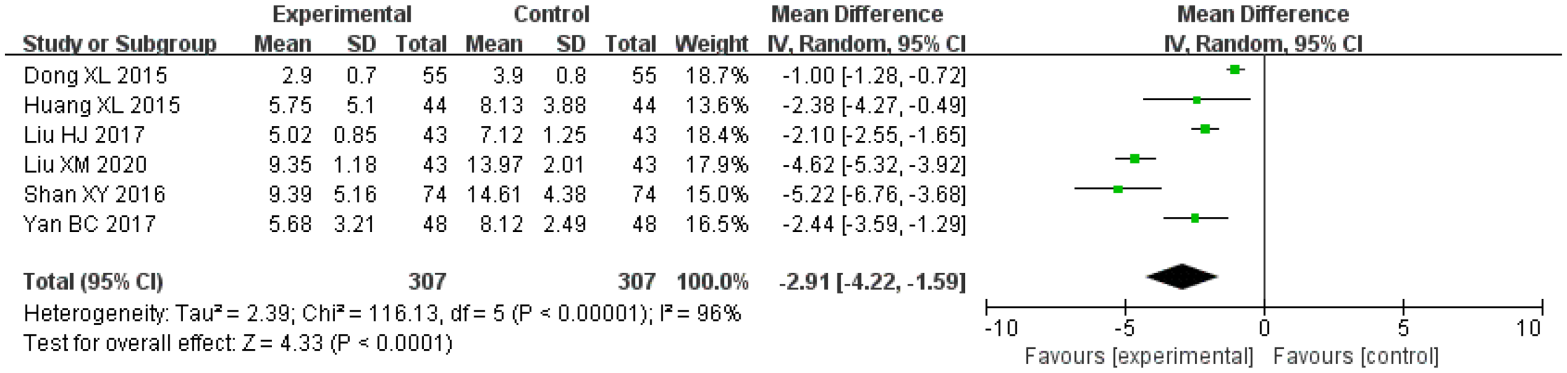


#### 3.5.2. mRS

A total of two studies on salvianolic acid assessed the mRS score and included 92 patients in the treatment groups and 92 patients in the control groups. There was no heterogeneity (*I*^2^ = 0%, *p* = 0.49) between the studies, and thus a fixed-effects model was applied for analysis. After combining the effect size, the results showed that the MD value was −0.73, with a 95% CI of −0.85 to −0.61; the result of the combined effect quantity hypothesis test showed that *Z* = 12.29 (*p* < 0.00001). This indicated that the observation group showed a lower degree of disability after treatment with Danshen preparations.


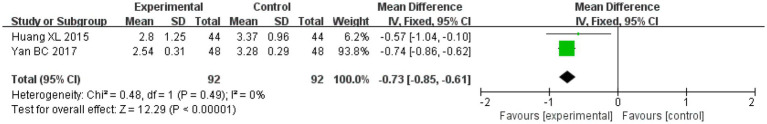


#### 3.5.3. BI

Amongst a total of three studies that reported on the BI, there were 135 cases in the treatment groups and 135 cases in the control groups. There was significant heterogeneity amongst the studies (*I*^2^ = 94%, *p* < 0.00001), and thus a random effects model was applied for analysis. After combining the effect size, the results showed that the MD value was 4.27, with a 95% CI of −19.50 to 28.04; the result of the combined effect quantity hypothesis test indicated that *Z* = 0.35 (*p* = 0.72). These results indicated that there were no significant differences in the ability to perform daily living activities (BI) between the observation group after treatment and the control group.


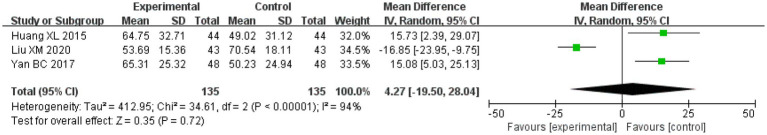


#### 3.5.4. ADL

A total of five articles and six studies [the article by Li et al. (2014), reported on two disease types and was thus considered to include two studies] assessed ADL. These studies included 342 patients in the treatment groups and 346 patients in the control groups. Two studies on salvianolic acid, three studies on tanshinone and one study on Danshen tablets. There was significant heterogeneity amongst the studies (*I*^2^ = 97%, *p* < 0.00001), and thus the random effects model was applied for analysis. After combining the effect size, the results showed that the MD value was 8.79, with a 95% CI of 3.52–14.06; the result of the combined effect quantity hypothesis test indicated that *Z* = 3.27 (*p* = 0.001). The results revealed that the post-treatment scores of life ability (ADL scale) in the observation group were significantly higher than those in the control group, indicating that after treatment with Danshen preparations, the patients exhibited significant improvement in their life ability.


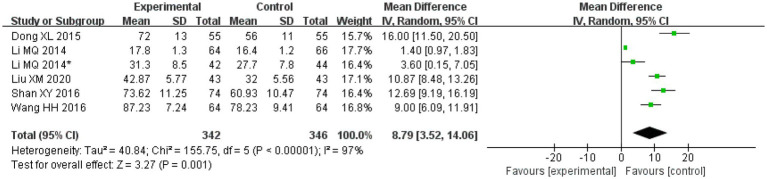


#### 3.5.5. MMSE

Amongst the included studies, a total of eight articles and nine studies [in the study by Li et al. (2013), patients were divided into two different groups and two outcomes were analysed, so the article was considered to consist of two studies], evaluated MMSE. There were 380 cases in the treatment groups and 380 cases in the control groups. The heterogeneity amongst the studies was observed to be significant (*I*^2^ = 93%, *p* < 0.00001), so the random effects model was applied for analysis. After combining the effect size, the results showed that the MD value was 2.09, with a 95% CI of 0.33–3.84; as per the result of the combined effect quantity hypothesis test, the *Z* was 2.33 (*p* = 0.02). These results indicated that the cognitive function (MMSE) score of the observation group after treatment was significantly higher than that of the control group. This suggests that treatment with Danshen preparations can improve the cognitive function of patients with VCI.


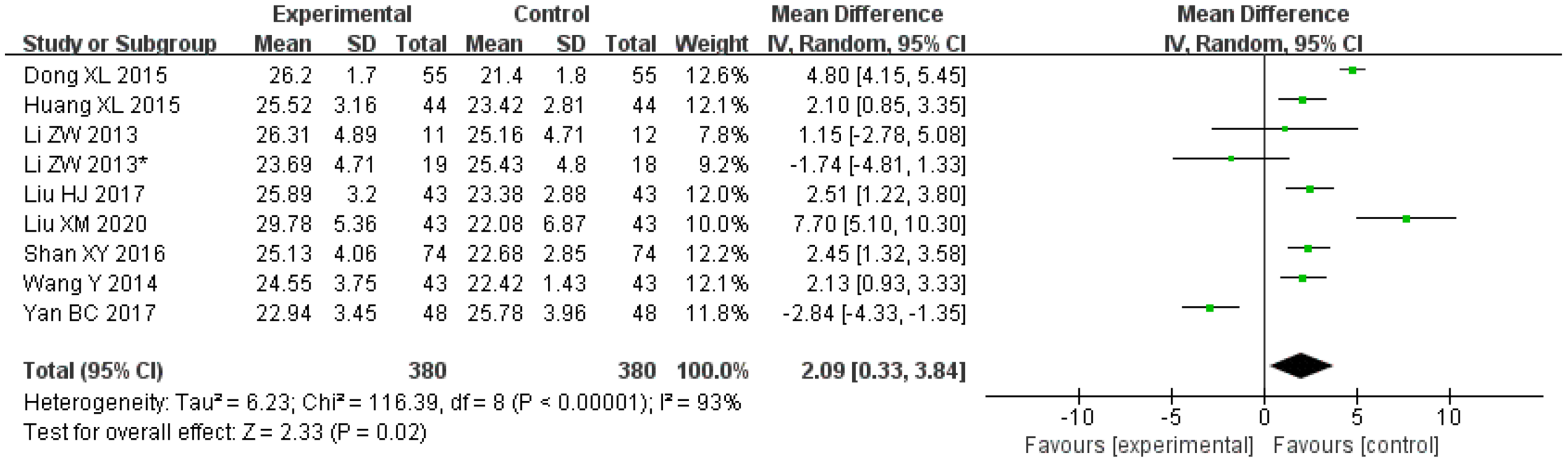


#### 3.5.6. MOCA

Amongst the included publications, a total of eight articles and nine studies [in the study by Li et al. (2013), patients were divided into two different categories and two outcomes were assessed; therefore, the article was considered to consist of two studies] evaluated MOCA scores. These included 334 cases in the treatment groups and 338 cases in the control groups. The heterogeneity amongst the studies was significant (*I*^2^ = 93%, *p* < 0.00001), so the random effects model was applied for analysis. After combining the effect size, the results showed that the MD value was 1.35, with a 95% CI of −0.50 to 3.20; the result of the combined effect quantity hypothesis test revealed that *Z* = 1.43 (*p* = 0.15), indicating that there was no significant difference in the score for post-treatment cognitive assessment (MOCA) between the observation group and the control group.


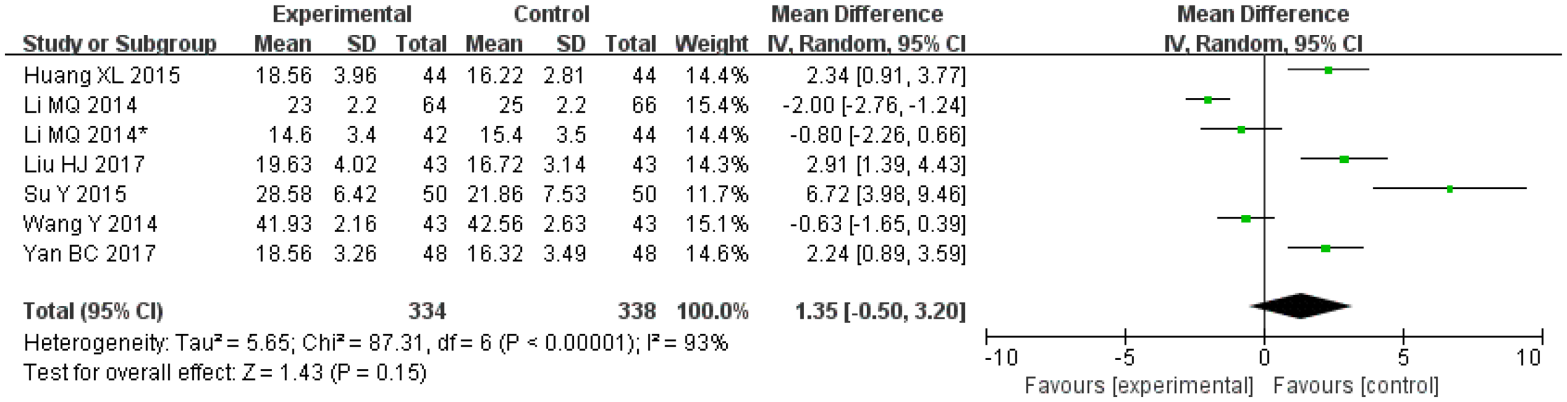


#### 3.5.7. HDS-R

There were 107 patients in the treatment group and 107 patients in the control group in the two included studies that evaluated the HDS-R. The heterogeneity between the two studies was significant (*I*^2^ = 99%, *p* < 0.00001), so the random effects model was applied for analysis. After combining the effect size, the results showed that the MD value was 3.66, with a 95% CI of −0.88 to 8.19, and the result of the combined effect quantity hypothesis test revealed that *Z* = 1.58 (*p* = 0.11). This indicated that there was no significant difference between the observation group and the control group in the cognitive assessment and intelligence test scores after treatment.


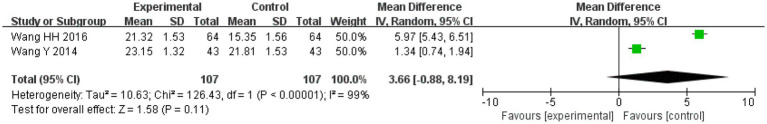


### 3.6. Inflammatory factors

#### 3.6.1. hs-CRP

A total of two studies on salvianolic acid and tanshinone evaluated hs-CRP levels, and included 98 patients in the treatment groups and 98 patients in the control groups. The heterogeneity between the studies was significant (*I*^2^ = 72%, *p* = 0.06), so the random effects model was applied for analysis. After combining the effect size, the results showed that the MD value was −3.21, with a 95% CI of −4.21 to −2.22, and the result of the combined effect quantity hypothesis test revealed that *Z* = 6.31 (*p* < 0.00001). This indicated that the hs-CRP levels in the observation groups were significantly decreased after treatment.


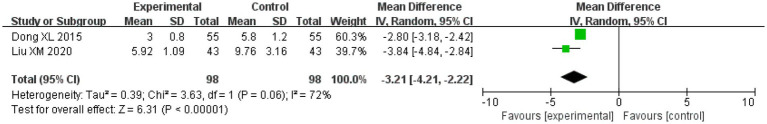


#### 3.6.2. IL-6

A total of two studies on salvianolic acid and tanshinone assessed the IL-6 levels amongst 98 patients in the treatment groups and 98 patients in the control groups. The heterogeneity between the two studies was found to be significant (*I*^2^ = 93%, *p* = 0.00002); thus, the random effects model was applied for analysis. After combining the effect size, the results showed that the MD value was = −2.95, with a 95% CI of −3.86 to −2.04; the combined effect quantity hypothesis test indicated that *Z* = 6.36 (*p* < 0.00001). This showed that the level of IL-6 in the observation group was significantly decreased after Danshen treatment.


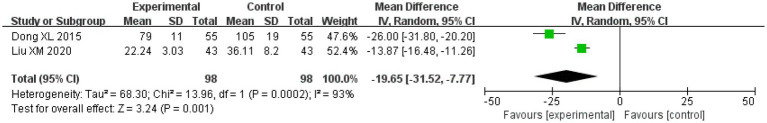


#### 3.6.3. TNF-α

A total of two studies reported on TNF-α levels; these studies included 98 patients in the treatment groups and 98 patients in the control groups. The heterogeneity between the studies was significant (*I*^2^ = 99%, *p* < 0.00001). Therefore, a random effects model was applied for analysis. After combining the effect size, the results showed that the MD value was −4.98 with a 95% CI of −12.77 to 2.81, and the result of the combined effect quantity hypothesis test showed that *Z* = 1.25 (*p* = 0.21). This indicated that there was no significant difference in the post-treatment TNF-α levels between the observation groups and the control groups.


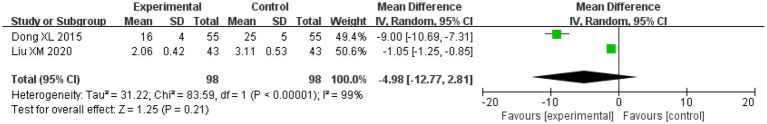


In summary, our analysis showed that treatment with Danshen preparations ameliorated inflammation in VCI patients to a certain extent.

### 3.7. Adverse reactions

Amongst the included studies, a total of four studies analysed adverse reactions, one study on salvianolic acid, two studies on tanshinone and one study on Danshen tablets, and included 261 patients in the treatment groups and 265 patients in the control groups. The heterogeneity amongst the studies was acceptable (*I*^2^ = 0%, *p* = 0.28). Therefore, a fixed-effects model was applied for analysis. After combining the effect size, the results showed that the OR value was 1.02, with a 95% CI of 0.48–2.15, and the combined effect quantity hypothesis test revealed that *Z* = 0.05 (*p* = 0.96). This indicated that there was no significant difference in the incidence of adverse reactions between the two groups.


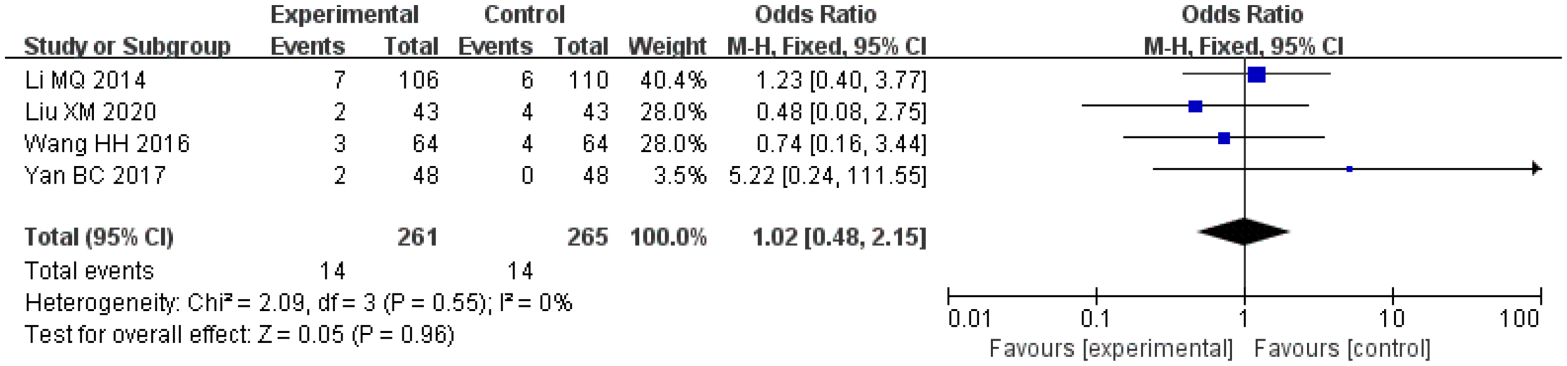


## 4. Discussion

The existing clinical intervention approaches for VCI can be categorised into two types: drug-based and non-drug-based. Non-drug-based therapy includes cognitive behavioural therapy, cognitive sandplay therapy, and cognitive dialectical behaviour therapy. Drug-based interventions include treatment with donepezil, which is a hexahydro pyridine derivative and a second-generation highly selective acetylcholinesterase inhibitor (Fan et al., 2020). This drug can specifically inhibit acetylcholine-degrading enzymes in the brain, and exhibits a bioavailability of 100% for oral formulations. Oxiracetam is a nootropic drug. This drug also improves cognitive dysfunction; it can cross the blood–brain barrier and is reported to promote the recovery of cerebral cortex and hippocampus nerve cells and ameliorate nerve blocks and metabolism (Shuster, 2011; Wang et al., 2020). Piracetam can act directly on the cerebral cortex to activate, protect, and repair brain nerve cells. It is mainly applied in treating memory loss and mild to moderate brain dysfunction. Piracetam can also prevent damage to the brain function caused by physical and chemical factors, improve reverse amnesia caused by ischaemia, effectively enhance memory retention, and improve learning ability. Vinpocetine can increase the oxygen content of brain tissue fluid in patients. It can improve the activity of haemoglobin, induce haemoglobin to release a large amount of blood oxygen, reduce blood viscosity, and improve brain function. Neural stem cell transplantation is a novel method for the treatment of many neurological diseases, especially ischaemic cerebrovascular disease. Several studies have demonstrated that exogenous neural stem cell transplantation can improve neurological function in rats after ischaemia (Ye et al., 2005; Liu et al., 2007). However, this method has not yet been widely used clinically due to issues regarding the purification of neural stem cells, transplant rejection, and other ethical issues. At present, there is no single drug nor non-drug therapy that can achieve a satisfactory curative effect, and the clinical treatment of VCI urgently needs to be improved. Danshen preparations can be used as complementary therapy in this regard.

It has been reported that traditional Chinese medicines used for invigorating the blood and removing blood stasis can have curative effects in many ischaemic diseases (Wang et al., 2017). Danshen is the root and rhizome of the Lamiaceae plant *Salvia miltiorrhiza*. It is bitter and slightly cold in nature and is attributed to the heart and liver meridians. It facilitates blood circulation and removes blood stasis, dredging channel and alleviating pain, clearing the mind, eliminating vexation, cooling the blood, and eliminating carbuncle. The medicinal value of Danshen has been known in China for more than 600 years, and it is an important component of traditional Chinese medicine. *Southern Yunnan Materia Medica* records that Danshen can “nourish the heart, tranquillise and sedate the mind, and resolve poor memory, restlessness, palpitation, and insomnia.” The chemical components of Danshen include tanshinones, cryptotanshinones, and salvianolic acids. Tanshinones are amongst the main active ingredients, and include tanshinone IIA and salvianol. The main active monomers of salvianolic acids are salvianolic acid A and salvianolic acid B (Jiang and Shi, 2017). Tanshinone can promote the proliferation and differentiation of neural stem cells *in vitro* (Xia et al., 2003; Yu et al., 2004), promote the proliferation of neural stem cells in the brain of rats with transient forebrain ischaemia, and improve neural function. Tanshinone IIA has effects on a variety of blood coagulation factors (Su et al., 2019). It has been reported to inhibit atherosclerosis, improve vascular smooth muscle function and collateral circulation in the ischaemic myocardium, inhibit neutrophil activation, reduce free radical levels, inhibit platelet aggregation, prevent thrombosis, and improve neural cell viability. This compound can also improve the memory and cognitive function in patients, restore their daily living ability, and prevent dementia; it is also reported to possess glucose-and lipid-lowering abilities (Jiang et al., 2019). Tanshinone IIA has been reported to improve learning and memory functions in VD rats. Combined with mesenchymal stem cells (MSCs), it can attenuate tau phosphorylation, enhance the activity of the central cholinergic system, reduce the generation of reactive oxygen species (ROS), and significantly enhance the activity of total superoxide dismutase (T-SOD) in the rat hippocampus. In addition, it can inhibit the apoptosis of hippocampal neurons by up-regulating the expression of BCL-2 and down-regulating the expression of BAX (Kong et al., 2017; Deyan et al., 2021). Tanshinone IIA sodium sulfonate is a derivative of tanshinone IIA and has high water solubility and bioavailability. It has anti-inflammatory, antioxidant, and anti-apoptotic effects (Cai et al., 2016). Moreover, it can improve the total antioxidant capacity, increase the level of superoxide dismutase, lower the levels of pro-oxidative products, and reduce neurological damage (Liu et al., 2010).

Salvianol can inhibit the mRNA expression of IL-1β, IL-6, and TNF-α, and inhibit the expression of mammalian Ste20-like kinase 1 (MST1) and the downstream transcription factor p-FOXO3. Accordingly, it improves working memory in VD rats and inhibits lipopolysaccharide (LPS)-induced apoptosis of hippocampal neurons (Yang et al., 2016). Salvianolic acid B ameliorates memory impairment in VD rats by increasing the level of insulin-like growth factor-1 (IGF-1) and the expression of P-AKT. Treatment with this compound promotes the attainment of a more compact morphology of neurons in the CA1 region, and a reduction in the apoptosis of these neurons (Ma et al., 2017). Salvia polyphenolate is a salvianolate compound extracted from Danshen; *Salvia miltiorrhiza* magnesium acetate is the main component herein. There are several advantages to this compound, as follows: its active ingredients have been identified, its quality is easy to monitor, its toxicity and side effects are limited, and its curative effect is stable (Chen et al., 2017). Salvia polyphenolate can directly cross the blood–brain barrier and act on mitochondria, ion pumps and other targets by increasing cerebral blood flow in the ischaemic area. It also has other functions as follows: prevents platelet aggregation, regulates intracellular Ca^2+^ concentration, inhibits cell apoptosis; it further exhibits anti-inflammatory and antioxidant properties s (Mi, 2013). Animal experiments have also confirmed (Wang and Wu, 2018) that salvianolate can increase ATPase activity, improve energy metabolism, and reduce excitatory amino acid content, thereby reducing cytotoxic oedema after cerebral ischaemia in mice; it is also reported to improve brain function, spatial learning, and memory. Additionally, salvianolate can decrease error rates in the step test, increase the delay time in the step test, and reduce the content of malondialdehyde (MDA) in the cortex, hippocampus, and striatum. This indicates that it can ameliorate the learning and memory impairment caused by cerebral ischaemia in mice (Wang, 2018).

This study used clinical efficacy, NIHSS, mRS, ADL, and MMSE as the main indicators of the effectiveness of Danshen preparations in VCI patients. According to the results of our MA, Danshen preparations have a positive effect in VCI patients, and can significantly improve neurological function and daily living ability. The NIHSS is the most widely used assessment tool for the predictive and prognostic evaluation of elderly patients with ischaemic cerebrovascular disease. This tool evaluates various parameters that reflect the neurological impairment of the patient including the patient’s state of consciousness, language skills, and motor status; this tool has good reliability and practicability (Hilz et al., 2011). The mRS is used to evaluate the recovery of neurological function in patients after a stroke. It has the advantage of providing strongly objective results and is not affected by education level (Weimar et al., 2002); thus, this scoring method overcomes the shortcomings and strong subjectivity of the NIHSS scale. The MMSE, developed by Folstein et al. (1975), is widely used in cognitive function screening and mainly evaluates functions such as orientation, memory, language, calculation, and attention. The MMSE takes a short time to complete and does not require a high education level. It has high sensitivity for evaluating cognitive impairment in moderate to severe dementia and impairment of multiple cognitive domains. MOCA, developed by Nasreddine et al. (2005), has a high screening sensitivity for cognitive impairment. It is designed to assess mental strength and mobility, but is also effective in assessing memory, language, thinking styles, and numeracy.

TNF-α, IL-6, and hs-CRP are commonly used indicators to assess the severity of inflammatory responses, and are considered to be closely related to the occurrence and development of VCI and VD (Zhang et al., 2020). IL-6 is a cytokine with various biological activities, and is an important inflammatory factor in the cytokine network. The abnormal release of this cytokine leads to metabolic disorders and functional impairment of cells in multiple organs of the body (Lu et al., 2000). hs-CRP is synthesised by stem cells in response to inflammatory stimuli such as microbial invasion or tissue damage, and is a non-specific phase protein secreted in the acute phase. It is commonly used as an indicator of the severity of inflammatory responses (Wang et al., 2015). hs-CRP participates in inflammatory responses and causes tissue damage by activating the complement pathway; it also promotes thrombosis. It has been reported that the measurement of hs-CRP levels at admission has an independent prognostic value in evaluating neurological deficits (Di Napoli et al., 2005); If inflammation persists, the protective effects evident in the acute phase may be converted to pro-inflammatory effects. Kuo et al. (2005) report that inflammatory markers can reflect the mechanism of cerebrovascular disease related to cognitive impairment, and changes in the levels of these markers can often be detected before the clinical symptoms of cerebrovascular disease appear. Elevated baseline hs-CRP levels suggest a direct toxic effect on neurons caused by long-term chronic inflammatory processes. The possible pathological mechanisms of hs-CRP include vascular damage caused by atherosclerosis, which further leads to frontal lobe circulation disorders, thereby affecting cognitive function (Liu, 2015). Inflammatory markers were used as secondary indicators in this study. Our MA suggests that Danshen preparations can alter the levels of the above-mentioned inflammatory markers associated with VCI. It is well known that cardiovascular diseases and tumour diseases are mostly caused by inflammation, and the anti-inflammatory activity of Danshen preparations can be widely used in the treatment of cognitive impairment patients with cardiovascular diseases or tumour diseases.

With the wide application of Danshen preparations in clinical practice, the number of reports of adverse reactions have increased. There is a lack of evidence-based studies that evaluate the efficacy and safety of Danshen preparations. This MA provides a comprehensive and complete reference for the clinical use of Danshen preparations. Our analysis showed that there was no significant difference in the occurrence of adverse reactions between Danshen preparation-treated and untreated groups. We believe that advancements in preparation technology have led to stricter quality control standards; which has led to a reduction in the occurrence of adverse reactions. However, it is also prudent to avoid the combined use of Danshen preparations with anticoagulants such as aspirin and warfarin, and strictly control the dose within a reasonable range.

### 4.1. Study limitations

This study provides a reference for clinical practice, but it also has certain limitations. Firstly, regardless of the quality of the included articles, the number of articles is small, and the overall sample size is also small. Secondly, there were differences in the randomisation methods used in the 12 included articles. Some studies used random number methods for randomisation; however, none of the articles specified that blinding was implemented. Thirdly, none of the included trials were registered as clinical trials, which may have affected the findings to some extent. Furthermore, most of the included trials lacked safety assessment protocols. Finally, differences in the drugs and courses of treatment administered as part of the basic treatment regimen may have led to biased results, which would affect the reliability of our findings.

## 5. Conclusion

This study reinforces the evidence that Danshen preparations have a significant therapeutic effect on VCI patients. Such preparations may lower inflammation and have beneficial effects on cognitive function in this patient population. Though our current study has some limitations, it is expected that further clinical trials will improve the level of evidence through stricter experimental designs, more comprehensive indicators, and by including large sample sizes.

## Author's note

MW (1966–), male, chief physician, doctoral supervisor, director of encephalopathy centre, Jiangsu Provincial Hospital of Chinese Medicine. Member of Stroke Society of Jiangsu Medical Association, deputy director of encephalopathy Professional Committee of Jiangsu Chinese Medicine Association. Standing committee member of Integrated Chinese and Western Medicine Branch of Chinese Stroke Association, Standing committee member of Integrated Chinese and Western Medicine Neurology of Chinese Medical Doctor Association, Member of Interventional Neurology of Chinese Research Hospital Association, visiting Scholar, Royal Melbourne Hospital, Australia.

## Author contributions

YL and YY: conceptualisation, visualisation, and methodology. XC and NY: data curation and investigation. YL and NY: formal analysis. YL and YY: writing–original draft. YL and NY: developed pictures. YY and XC: developed tables. JL: writing– review and editing. MW and JL: supervision. All authors contributed to the article and approved the submitted version.

## Funding

Project of Jiangsu Provincial Hospital of Chinese medicine (Y18004, Y19044, Y20025, and Y2019CX10); The Open Projects of the Discipline of Chinese Medicine of Nanjing University of Chinese Medicine Supported by the Subject of Academic priority discipline of Jiangsu Higher Education Institutions no. ZYX03JG035.

## Conflict of interest

The authors declare that the research was conducted in the absence of any commercial or financial relationships that could be construed as a potential conflict of interest.

## Publisher’s note

All claims expressed in this article are solely those of the authors and do not necessarily represent those of their affiliated organizations, or those of the publisher, the editors and the reviewers. Any product that may be evaluated in this article, or claim that may be made by its manufacturer, is not guaranteed or endorsed by the publisher.

## Figures and Tables

**Table:** 

VCI	vascular cognitive impairment
CNKI	China National Knowledge Infrastructure
CQVIP	Chongqing VIP Database
CBM	China Biology Medicine
NIHSS	National Institutes of Health Stroke Scale
mRS	modified Rankin Scale
BI	Barthel Index
MMSE	Mini-Mental State Assessment
MCA	Montreal Cognitive Assessment
ADL	Activities of Daily Living Scale
MA	meta-analysis
CI	confidence interval
IL	interleukin
VD	vascular dementia
PICOS	Participants, Interventions, Comparisons, Outcomes and Study Design
BMI	body mass index
RR	risk ratio
MD	mean differences
MOCA	Montreal Cognitive Assessment
HDS-R	Hasegawa Dementia Scale
TNF	tumour necrosis factor
hs-CRP	high-sensitivity C-reactive protein
OR	odds ratio
MSCS	mesenchymal stem cells
ROS	reactive oxygen species
MST1	mammalian Ste20-like kinase 1
LPS	lipopolysaccharide
IGF-1	insulin-like growth factor-1
MDA	malondialdehyde
MD	mean difference
STAT3	signal transducer and activator of transcription 3
